# Engineered biochar nanocomposites: integrating nanotechnology with carbon-based materials to enhance plant resilience against biotic and abiotic stresses

**DOI:** 10.3389/fpls.2026.1775263

**Published:** 2026-04-29

**Authors:** Liaqat Ali, Natasha Manzoor, Temoor Ahmed, Aown Abbas, Muhammad Farhan Qadir, Chen Song, Gang Wang

**Affiliations:** 1 College of Resources and Environment, Xinjiang Agricultural University, Urumqi, China; 2 Department of Soil and Water Sciences, China Agricultural University, Beijing, China; 3 State Key Laboratory of Rice Biology and Breeding, Ministry of Agriculture Key Laboratory of Molecular Biology of Crop Pathogens and Insects, Key Laboratory of Biology of Crop Pathogens and Insects of Zhejiang Province, Institute of Biotechnology, Zhejiang University, Hangzhou, China; 4 Department of Plant Biotechnology, Korea University, Seoul, Republic of Korea; 5 Department of Life Sciences, Western Caspian University, Baku, Azerbaijan; 6 Department of Geography and Resource Management, The Chinese University of Hong Kong, Hong Kong, Hong Kong SAR, China; 7 Institute of Soil and Environmental Sciences University of Agriculture Faisalabad, Faisalabad, Pakistan

**Keywords:** abiotic stress, biotic stress, climate change, engineered biochar, nanocomposites, nanotechnology, sustainable agriculture

## Abstract

Climate change intensifies plant stress factors and threatens global food security and therefore demands sustainable land management practices. Engineered biochar nanocomposites (EBNCs) are the synergistic combination of nanotechnology to improve carbon sequestration and sustainable approach for the precise functionality of biochar (BC) to amend soil properties. This comprehensive review examines the design, fabrication, and mechanisms of EBNCs to mitigate both biotic and abiotic stresses in plants. We analyze recent advances in EBNC synthesis through physical, chemical, and biological integration routes for incorporating metal/metal oxide nanoparticles (NPs) i.e., Ag, Fe, ZnO, TiO_2_, and graphene into BC matrices. EBNCs induced mechanisms of stress alleviation are enhanced water retention, ion homeostasis, heavy metal (HM) immobilization, antimicrobial activity, induced systemic resistance (ISR), and scavenging reactive oxygen species (ROS). The applications of EBNCs significantly improve crop performance under drought, salinity, HM contamination, or pathogen stress. However, production costs, environmental trade-offs, potential toxicity, and regulatory policies are research gaps and require further careful consideration. Future research should focus on developing smart, stimuli-responsive EBNCs with controlled-release properties, integration of omics and artificial intelligence tools for optimized formulations, and circular economic approaches. EBNCs are increasingly recognized as multifunctional materials with potential applications in climate-resilient and sustainable agriculture.

## Graphical abstract

**Figure:**
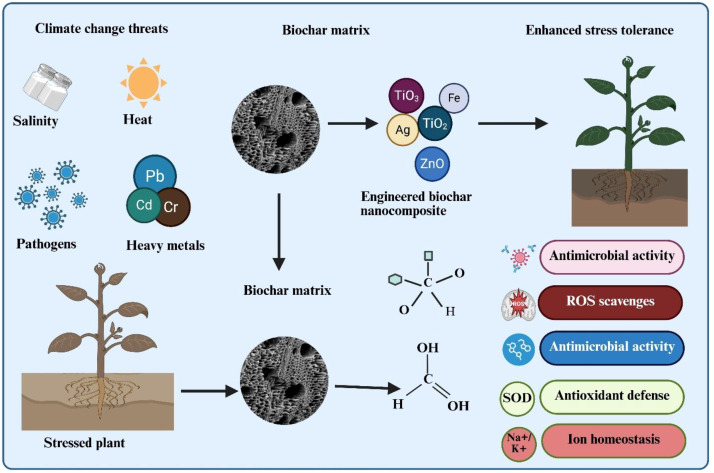

## 1 Introduction

**Figure 1:**
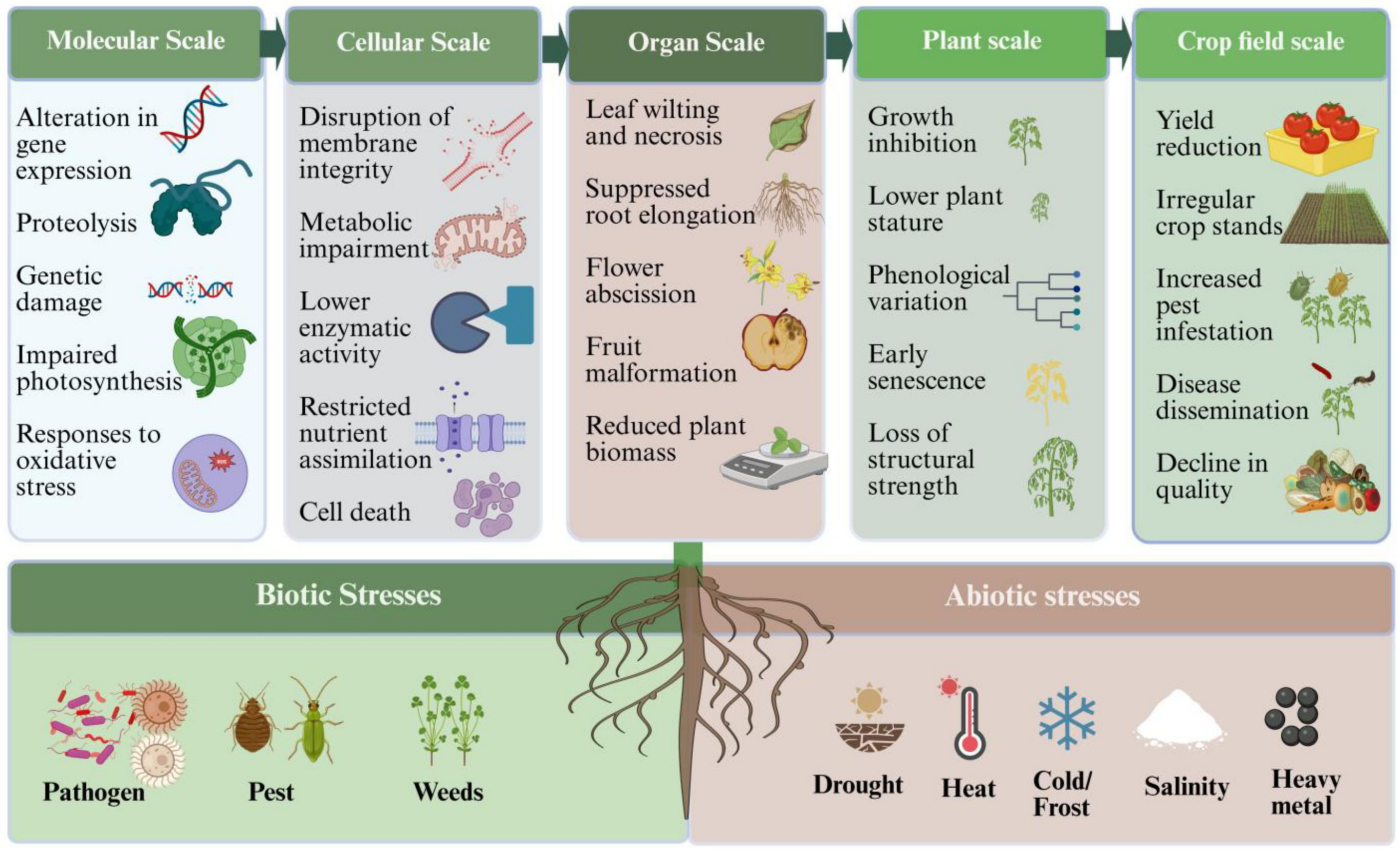
Multi-scale impacts of biotic and abiotic stresses on crops: From molecular disruption to field-level yield loss. The image was created using BioRender (biorender.com).

Global food security is increasingly threatened by climate change, which is expected to intensify both biotic and abiotic stresses on crops, thereby reducing yield and affecting agricultural productivity (Atanda et al., 2025). World projections for 2100 suggest that crop yield under the business-as-usual scenario (SSP5-8.5) may decrease by 22% maize, 15% soybean, 14% wheat and 9% rice. In vulnerable regions including sub-Saharan Africa, North America and South Asia, food supplies will be 15-30% calorie deficits due to this climate change (Li et al., 2025). Soil degradation from pesticide and fertilizers, rising temperatures and erratic weather patterns, increases in salinity and increasing pressure from pathogens collectively threaten the sustainability of food production systems (Rahim et al., 2025) for future generations. The loss of crops from abiotic stress such as drought, salinity, heavy metals (HMs) and temperature ranges as well as biotic stress causes from pathogens, plant pests and weeds, can exceed 50% worldwide annually (Gao et al., 2025). Biotic and abiotic stresses induce cascading effects across multiple biological scales, from molecular and cellular disruptions to whole-plant dysfunction and field-level yield losses (**Figure 1**). According to the Global Food Crisis Report 2024, there are more than 95.8 million people who have been displaced forcibly from their homes primarily due to conflict-driven agricultural disruption and/or due to their country’s climate change related loss to agricultural production (Liu et al., 2025). Traditional mitigation strategies are effective; however, they tend to deal with a single stressor and lack the multifunctionality and precision of dealing with multiple stressors using sustainable holistic practice.

The convergence of nanotechnology and carbon-based products has opened new frontiers in agricultural science, particularly through the development of engineered biochar nanocomposites (EBNCs) that blend the inherent qualities found in biochar (BC) with the advanced precision and capacity of engineered nanomaterials (Minello et al., 2025). Presently, most methods of BC application do not provide the required precision or focus when it comes to mitigating plant stress. As traditional BC has some limitations including limited capacity to provide nutrients in a timely and precise manner, the release of nutrient is generally not predictable, leading to significant losses from leaching (Luo et al., 2024), inconsistent performance of BC across different soil types and environment (Bo et al., 2023), its ability to produce targeted plant response to stress is limited due to the broad spectrum nature of commercial BC (Mukherjee and Lal, 2014; Tsolis and Barouchas, 2023; Boydak et al., 2025), and relatively slow response times for stress mitigation (Atkinson et al., 2010), especially under acute stress conditions. Furthermore, whereas release pattern of nutrients from conventional BC is delayed relative to the plant developing sudden stress, as evidence by the difference in nutrient release pattern between sandy and clay soils (Shackley et al., 2010). Using engineered nanomaterials allows for the development of hybrid BC, which represents an alternative and potentially improved product to mitigate plant stressors, producing benefits such as controlled release of nutrients, increased surface area for chemical reactivity and stress targeting mechanisms (Elhakem et al., 2025). Thus, compared to conventional BC, EBNCs have been proposed as an approach to address some of the above-mentioned limitations of traditional BC by integrating created properties into BC via engineered nano-scale materials; thereby allowing for increased bioavailability of nutrients to plants and improved specificity of plant responses to mitigate stressors (Wang et al., 2017). The synergistic combination of EBNCs overcomes the temporal and spatial limitations of traditional BC and enables adaptive response capabilities (Ali et al., 2024). In addition, EBNCs enhance adaptive capacity to changes and are able to respond to multiple forms of stress at once and to do so in an environmentally sustainable manner. EBNCs offer efficient and engineered solutions with control of nutrient release rates, enhance plant-BC interactions, and multiple stress mitigation mechanisms (Kamal et al., 2025). These EBNCs materials exhibit enhanced water retention ability, provide greater and better-balanced nutrient delivery, provide antimicrobial properties, and modulate plant physiology in response to stress (Sana et al., 2024). As complexity and uncertain characterize the agri-food industry, EBNCs may become valuable tools for improving resilience and supporting sustainable food production systems under changing climate conditions (Singh et al., 2024).

While previous reviews have addressed BC and agricultural nanomaterials separately or offered descriptive accounts of their combined use (Kah et al., 2019; Manzoor et al., 2024), key questions regarding synergistic mechanisms and rational EBNC design remain unresolved. This review highlights emergent properties of EBNCs absent in individual components, including stress-responsive nanoparticle (NP) release, dual-phase ROS regulation, and coordinated rhizosphere-level mitigation of abiotic stresses (Mittler, 2017; Kah et al., 2019; Tsolis and Barouchas, 2023). We further synthesize evidence that EBNCs comparatively co-regulate multiple stress-response pathways and highlight mechanistic design considerations such as NP-matrix compatibility, threshold-responsive engineering, and BC–NP complementarity that enable predictive, stress-specific EBNC development (Kah et al., 2019; Raza et al., 2023; Tsolis and Barouchas, 2023).

This review provides a critical, mechanistic synthesis of EBNCs as next-generation agricultural materials, with a specific focus on how matrix–NP interactions generate emergent, non-additive functionalities that enhance plant resilience under multiple stressors. We examine current EBNC synthesis and characterization strategies, dissect stress-specific biological mechanisms across biotic and abiotic contexts, and evaluate translational considerations including field performance, scalability, and commercialization barriers. By integrating pathway-level analyses with structure–function relationships, this review moves beyond descriptive summaries to offer design-oriented insights that guide rational EBNC formulation and application. The perspectives presented here aim to support researchers, industry stakeholders, and policymakers in advancing EBNCs from laboratory-scale concepts toward field-ready, climate-resilient technologies that contribute to sustainable agriculture, global food security, and environmental stewardship.

## 2 Biochar and nanotechnology: the foundation of EBNCs

BC obtained through the pyrolysis of organic matter under limited oxygen availability, has become a widely used product due to its many benefits as an addition to soil that also sequesters carbon over time (Ayaz et al., 2025). The wide range of uses BC is due to its exclusive features such as having a very porous structure (with large surface area) and having many types of chemical functional groups which permit interaction with soil constituents, nutrients and plant roots (Arshad, 2024). BC also maintains long-term storage of carbon due to its durability and improves soil physicochemical and biological properties of soil. While BC has been shown to assist plants in dealing with stress through its effect on water retention, nutrient availability, soil acidic or alkaline pH, and serving as a favorable habitat for beneficial microbes (Chandi et al., 2024). However, there are limitations to conventional applications of BC regarding the facility to deliver BC to specific areas of need, to control the rate of delivery (i.e., slow release), and address specific BC mechanisms for stress alleviation in growing plants (Tikoria et al., 2023). These limitations have driven the development of new methods to improve the functionality of BC by incorporating nanotechnology into the engineering process that create the final BC product (Yang et al., 2025).

Nanotechnology provides a precision tool for agricultural purposes through materials typically sized below 100 nm (nanometers) exhibiting exceptional physical, chemical and biological properties compared to normal bulk materials. Such nano-enabled strategies have been applied to enhance crop nutrition, protection, and stress tolerance (Kah et al., 2019; Demirkıran and Sohrabi, 2024; Jafir et al., 2024). Metal/metal oxide NPs such as iron (Fe), silver (Ag), zinc oxide (ZnO), titanium dioxide (TiO_2_), and carbon-based nanomaterials (such as graphene) have shown great potential for stress alleviation in plants (Manzoor et al., 2021; Sehrish et al., 2024; Zhang et al., 2024). The way by which NPs help plants against stresses includes enhancement of antioxidant enzymes system, direct antimicrobial activity, enhancement in nutrient absorption and modulation of gene expression (Abdelhameed et al., 2025). Recent studies also indicate that through their effect on plants, NPs may stimulate the plants own ability to induce systemic resistance (ISR), a type of natural defense against various stress factors (Kalia and Sreelakshmi, 2025). The distinctive properties of NPs are directly related to size, which allow them to be used as target delivery and improve bioavailability of NPs compared to their larger forms.

EBNCs represent a new approach in strategically combining NPs together with a BC matrix to produce a bioproduct that utilizes nanotechnology for precise enhancement of its functional properties (Kumar et al., 2024). By combining the properties of both NPs and BC, the synergy between both of these components allows for greater functionality than either would have on its own. The BC structure serves as physical carrier for the NPs during application, this prevents clustering of the NPs as well as allows for controlled release (Viswanathan et al., 2024). The distinctiveness of EBNCs is that they possess multiple functional benefits at once, including carbon sequestration, nutrient delivery, improved soil quality, and minimization of plant stress under adverse growing conditions (Shahzadi et al., 2025). Current research indicates that EBNCs may provide greater benefits than BC used alone or NP applied individually, thereby supporting the positive effects of synergies (Ali et al., 2024). However, methods used to combine NPs with BC can be designed to suit individual agricultural needs, crop requirements and environmental conditions.

### 2.1 Emergent properties of EBNCs

EBNCs exhibit emergent, non-additive properties that differentiate them from physical mixtures of BC and NPs. These arise from matrix–NPs interactions that influence NPs retention, passive release kinetics, enhance NP stability, and influence biological regulation. Unlike BC (passive diffusion) or bare NPs (rapid release and aggregation), EBNCs provide controlled, sustained delivery through pore confinement and surface functionalization, extending activity from days to weeks while reducing aggregation and leaching (Ghassemi-Golezani and Rahimzadeh, 2022; Aborisade et al., 2023; Manzoor et al., 2024). Matrix–NPs interactions primarily influence NPs retention and passive release kinetics through adsorption–desorption equilibria and pore confinement. It is important to distinguish these diffusion-controlled release mechanisms from truly stimuli-responsive systems incorporating stress-labile chemical linkages (e.g., pH-, ROS-, or enzyme-sensitive bonds), which remain comparatively rare in current EBNC formulations. Oxidative stress conditions may influence the functional behavior of EBNCs, potentially linking plant stress intensity with changes in material performance; however, such as adaptive feedback mechanisms remain to be systematically validated across crop–soil systems (Manzoor et al., 2024). Several studies suggest that EBNCs may facilitate ROS homeostasis by simultaneously enhancing antioxidant capacity while maintaining basal ROS signaling required for stress adaptation (Hasanuzzaman et al., 2020; Aborisade et al., 2023). However, this proposed dual-phase modulation remains an emerging mechanistic hypothesis rather than a universally demonstrated EBNC-specific phenomenon, as most studies infer ROS regulation from antioxidant enzyme activity and lipid peroxidation markers rather than direct temporal ROS quantification. Furthermore, EBNCs spatially co-localize water retention, nutrient supply, metal immobilization, and antimicrobial activity within rhizosphere microenvironments, generating multifunctional “stress refuge zones” that outperform single-function amendments (Aborisade et al., 2023; Chandi et al., 2024). Simultaneously, BC matrices mitigate environmental risks by limiting NPs accumulation in plant tissues and non-target exposure, effectively converting NPs from potential liabilities into controllable agricultural assets (Ahmad et al., 2024). Collectively, these observations suggest that EBNCs can exhibit synergistic behavior beyond simple additive combinations under defined experimental conditions. Nevertheless, the extent and reproducibility of such emergent effects remain dependent on formulation parameters, stress intensity, and environmental context.

## 3 Design and fabrication of engineered biochar nanocomposites

### 3.1 Methods of integration: physical, chemical, and biological routes

The fabrication of EBNCs involves various methods, each with distinct advantages and associated limitations. The physical methods include mixing, coating and impregnation of NP onto BC while keeping both components properties intact (Zhou et al., 2024). The methods are relatively straightforward to implement and can be manufactured at the low cost, making them beneficial to large scale production (Viswanathan et al., 2024). However, the weak bonding (interactions) between the BC and NPs limits the performance of these types of EBNCs. On the other hand, there are chemical methods to create EBNCs such as co-precipitation, functionalization and *in-situ* synthesis (Hassan et al., 2025). These methods will create stronger interfacial bonds between the two components, thus making them more stable and having greater capabilities. However, they do have the potential to use more complicated processing conditions and potentially hazardous materials (Khalid et al., 2023). Advanced chemical methods enable precise control over NPs distribution, size, and surface properties. The biological methods of making EBNCs represent a developing area for creating NPs, in which microorganisms or biological processes create the NPs directly on the surface of the BC (Deng et al., 2023). The approaches using biological synthesis to create NPs are cost effective, environmentally friendly and reduce the use of potentially harmful materials (Kalidasan et al., 2025). In addition to reduced chemical inputs, biologically synthesized NPs may exhibit enhanced biocompatibility and lower ecotoxicological risks. It is important to note that most currently reported EBNC fabrication approaches yield systems governed by passive release mechanisms, primarily controlled by pore architecture, adsorption–desorption equilibria, and diffusion constraints. Although such systems are sometimes described as “controlled,” their release behavior typically reflects physicochemical interactions rather than engineered trigger-dependent activation. In contrast, truly stimuli-responsive EBNCs require incorporation of stress-labile chemical linkers, redox-sensitive bonds, or enzyme-cleavable coatings that undergo structural transformation under defined environmental stimuli. Such architecture remains comparatively rare in agricultural EBNC applications and represents an important direction for future material design.

### 3.2 Types of nanomaterials, green synthesis and biocompatibility considerations

BC, which contains silver nanoparticles (AgNPs), has strong antimicrobial effects against numerous plant pathogens while at the same time being biocompatible. Studies have shown that BC that contains Ag has a longer lasting antimicrobial effect and releases less Ag than AgNPs alone (Bytešníková et al., 2022; Irshad et al., 2024). Due to the BC matrix, silver ions Ag^+^ are released in a controlled manner, allowing for sustained protection, and reducing the threat of toxicity. Fe-based nanocomposites have demonstrated great success in remediating heavy metals and reducing oxidative stress (Manzoor et al., 2021). Fe-BC composites have been shown to enhance antioxidant enzyme activity, decrease levels of reactive oxygen species (ROS) and immobilize heavy metals via many different pathways (Mahmoud et al., 2024). The magnetic properties of iron nanoparticles (FeNPs) also have capacity to efficiently remove and possibly reuse composite material (Nikić et al., 2024). Zinc oxide nanoparticles (ZnONPs) combined BC provide antimicrobial activity and significantly enhanced plant growth and improved stress tolerance (Ahmad et al., 2024). These composite ensured adequate nutrition with controlled release of Zn while preventing toxicity (Sana et al., 2024). ZnO with photocatalytic characteristics can also aid in breakdown of organic pollutants in soil environments (Ghandali et al., 2024). In addition, distinct photocatalytic properties of titanium dioxide nanocomposites (TiO_2_NPs) trigger ROS generation for antimicrobial activity and enable degradation of organic pollutants (Sehrish et al., 2024). TiO_2_/BC have greater capability to remediate the soil under different environmental conditions and enhanced stress tolerance in plants compared to absence of TiO_2_/BC (Abdelhameed et al., 2025). Graphene oxide nanocomposite developed with high degree of electrical conductivity (EC), mechanically stronger having higher specific surface area. These carbon-based NCs can deliver nutrients, retain more water and increase EC when applied to plants (Mandal et al., 2025; Rafique et al., 2025). The compatibility between these carbon-based nanomaterials and BC enables stable integration with least environmental impact.

The green synthesis techniques applied for EBNC production promote environmental friendly and less toxic practices while utilizing sustainable production approaches (Viswanathan et al., 2024). Plant extract-mediated synthesis utilizes natural reducing agents and capping agents to produce NPs with higher biocompatibility (Mahmoud et al., 2024). These methods often result in NPs with exceptional chemical surfaces that allows interaction between the plant and NPs with reduced environmental risk (Kalidasan et al., 2025). In microbially mediated synthesis approaches, bacteria, fungi and/or other microorganisms employed to produce NPs directly on the surface of BC (Deng et al., 2023). This biological approach provide precise control across NPs properties while maintaining environmental safety requirements (Mushtaq et al., 2024). The origin of NPs through biological means increased their compatibility with plant and soil microorganisms. The evaluation of NPs specifically regarding biocompatibility consider not only their leaching in soil profile, their potential toxicity to plants and soil microorganisms but also their long-term impact on the environment (Zhang et al., 2024). Recent studies indicates that biocompatibility can be achieved through surface functionalization and controlled release mechanisms, while providing optimal functionality (Chandi et al., 2024). Thus, EBNCs design should achieve a balance between improving plant performance with potentially safe and sustainable environmental (Atanda et al., 2025).

### 3.3 Characterization techniques: understanding EBNC functionality

Comprehensive characterization of EBNCs requires multiple analytical techniques to elucidate their structural, functional and chemical characteristics. Such as scanning electron microscopy (SEM) and transmission electron microscopy (TEM) provide comprehensive knowledge about NPs size, distribution and their morphology within the BC matrix (Mahmoud et al., 2024; Sana et al., 2024). These imaging techniques aid in determining the optimal synthesis conditions for EBNCs and provide an insight into the structure-property relationships. Another critical tool for EBNCs characterization is X-ray diffraction (XRD), which help to determine the crystalline phases and structural changes in EBNCs as NPs are integrated. X-ray photoelectron spectroscopy (XPS) provides information about chemical composition of surface and oxidation state of EBNCs (Hassan et al., 2025). Fourier transform infrared spectroscopy (FTIR) assist in identifying the functional groups present on the surfaces and determine the interactions between NPs and BC (Nworie et al., 2024). Moreover, surface area and pore structure analysis through nitrogen adsorption-desorption isotherms that help to determine changes in surface area and porosity caused by the NPs integration (Latifian et al., 2024). And these properties have direct influence on water retention, nutrient adsorption and release characteristics of EBNCs. While thermal analysis techniques provide knowledge about thermal stability and degradation behavior under diverse environmental conditions (Zhou et al., 2024).

## 4 Mechanisms of EBNCs in mitigating abiotic stress

Engineered biochar-based nanocomposites (EBNCs) mitigate abiotic stresses through integrated physicochemical and biological mechanisms, including water retention, ion regulation, redox homeostasis, and contaminant immobilization (**Table 1**).

**Table 1:** Mechanisms and applications of biochar-based nanocomposites for mitigating crop abiotic stresses.

Crop	Stress type	EBNC type	Application result	Proposed mechanism	Reference
Tomato (*Solanum lycopersicum*)	Salinity	Nano-BCr colloidal solution (NBS)	Improved shoot and root length (53% & 36%), biomass and yield (fruit number & weight), total chlorophyll (53–72%), carotenoids (40–64%) and increased leaf RWC (2.5×). Reduced EL (28–30%), MDA (46%) and boost antioxidant enzymes & metabolites.	NBS stabilizes membranes, reduces oxidative damage by improving photosynthetic pigments and osmotic status, strengthens antioxidant defense	(Shahzadi et al., 2025)
Spinach (*Spinacia oleracea* L.)	Salinity	ZnO-NPS + BC	Enhanced growth, chlorophyll, gas-exchange and antioxidant enzyme activities. Reduced Na^+^ in roots (57.69%) and in leaves (61.27%). Higher N,P,K contents	ZnO NPs + BC reduces Na uptake/translocation, improves nutrient availability and boost antioxidant (SOD, CAT etc.)	(Ahmad et al., 2024)
Brassica *chinensis* L.	Heavy metal	Nanoscale zero-valent Fe + BC and activated carbon (nZVI-ESB/AC)	Higher immobilization of Pb & Cd. Reduced metal bioaccumulation in edible parts. Improved plant growth; oxidative-stress indicators reduced by (1.5–2×)	nZVI-ESB/AC stabilizes Pb & Cd in soil, supplies Fe that stimulates antioxidant enzyme activity, lower ROS and MDA level	(Aborisade et al., 2023)
common buckwheat (*Fagopyrum esculentum*)	drought	Rice husk BC and nano iron oxide	BC + nano-Fe_2_O_3_ markedly improved buckwheat growth, yield, and antioxidant activity under drought compared with individual applications	BC enhanced soil moisture and nutrient retention, while nano-iron improved Fe availability and ROS detoxification, jointly increasing drought tolerance	(Sah et al., 2025)
German chamomile (*Chamaemelum* *chamomilla* L.)	Salinity	MgO-BNP, Fe_3_O_4_-BNP and combinations (BNP-MgO, BNP-Fe_3_O_4_)	Enriched BNP treatments improved root and shoot mass, root/shoot ratio. BNP-Fe_3_O_4_ improved organ mass while BNP-MgO improved pigments. Enhanced essential oil constituents and production	BNPs reduced H_2_O_2_ (ROS) and modulated enzymatic/non-enzymatic antioxidant responses; improved nutrient cycling availability	(Solhi-Khajehmarjan et al., 2025)
chili peppers (*Capsicum annuum* L.)	Heavy metal	BC and nanoscale silicon	Combined BC + nanosilicon significantly reduced arsenic toxicity, enhanced antioxidant enzyme activity, improved growth, and increased yield of Capsicum annuum compared with single applications.	BC immobilizes arsenic and improves soil properties, while nanoscale silicon enhances antioxidant defense, membrane stability, and limits As uptake and translocation in plants.	(Manzoor et al., 2024)
Wheat	Drought	Nano-biochar (NBC) + brassinosteroids (BR)	increased plant height, spike length, fertile tillers, grains per spike, grain weight and yield (4079 kg/ha). Improved LAI, RWC, chlorophyll and N/P/K in grain. Trigger antioxidant enzyme activities	NBC improves soil water retention, NUE and stimulates growth regulators; reduces oxidative stress	(Raza et al., 2023)
rice (*Oryza sativa* L.)	Salinity	BC + ZnO and Fe_2_O_3_ NPs	Under saline conditions (EC ≈10.5 dS m^-1^), nano-engineered BC significantly improved rice growth, chlorophyll content, water status, and antioxidant enzyme activities while reducing oxidative damage	Nano-engineered BC improves K^+^/Na^+^ homeostasis, reduces Na^+^ toxicity, and enhances antioxidant defense, maintaining physiological stability under salinity	(Sultan et al., 2025)
Tobacco	Heavy metal	BC+ AM/CMC	Increased Cd adsorption and reduced toxicity. Increased root and shoot biomass, RWC, photosynthesis, stomatal density. Upregulate the antioxidant enzymes and lower decreased MDA and cell death, by lowering ROS	Composite reduced the Cd bioavailability thereby lowering plant Cd uptake. Enhanced antioxidant response to stress and lower oxidative damage	(Du et al., 2023)
Rice (*Oryza sativa*)	Heavy metal	BC + Ca/Mg nanocomposite	Enhanced improved plant growth, dry biomass, tillers, grains per tiller, SPAD value, total chlorophyll. Improved antioxidant enzymes and metabolites. Reduced Cd in roots, shoot and grains (30%, 33%, 40% respectively).	In combination composite immobilizes Cd and reduce bioavailability. Improve nutrient status, lower Cd accumulation and oxidative damage	(Ali et al., 2024)
Dill (*Anethum graveolens* L.)	Salinity	BC-nanocomposite: BNC-FeO, BNC-ZnO	BNCs reduced Na accumulation (22–25%), Increased plant Zn and Fe in leaves (91–118%), improved essential oil composition and antioxidant activity of seed essential oil (IC_50_ decreased; antioxidant capacity ↑ ~32–34% under high salinity).	BNCs improve nutrient (Fe/Zn) supply and reduce Na uptake, promote secondary metabolism and reduced salt oxidative damage.	(Ghassemi-Golezani and Rahimzadeh, 2022)
Wheat	Drought	BC and Si-NPs	BC+SiNPs enhanced wheat growth, yield, and antioxidant activity under drought more effectively than individual applications	BC improved soil moisture and nutrients, while SiNPs strengthened antioxidant defenses and reduced oxidative stress.	(Zulfiqar et al., 2024)
Sonchus oleraceus L. and *Myriophyllum verticillatum* L.	Heavy metal	SiO_2_-NP-modified biochar	Combined SiO_2_-NP–BC application significantly alleviated HM stress, improved physiological traits, boosted antioxidant enzyme activities, and enhanced plant growth compared to unmodified biochar or NPs alone.	SiO_2_-NP–modified BC immobilizes HMs in soil, reduces metal uptake, and enhances antioxidant defense and nutrient balance in Allium hookeri.	(Su et al., 2025)

### 4.1 Drought stress: enhancing water retention and ROS scavenging

**Figure 2:**
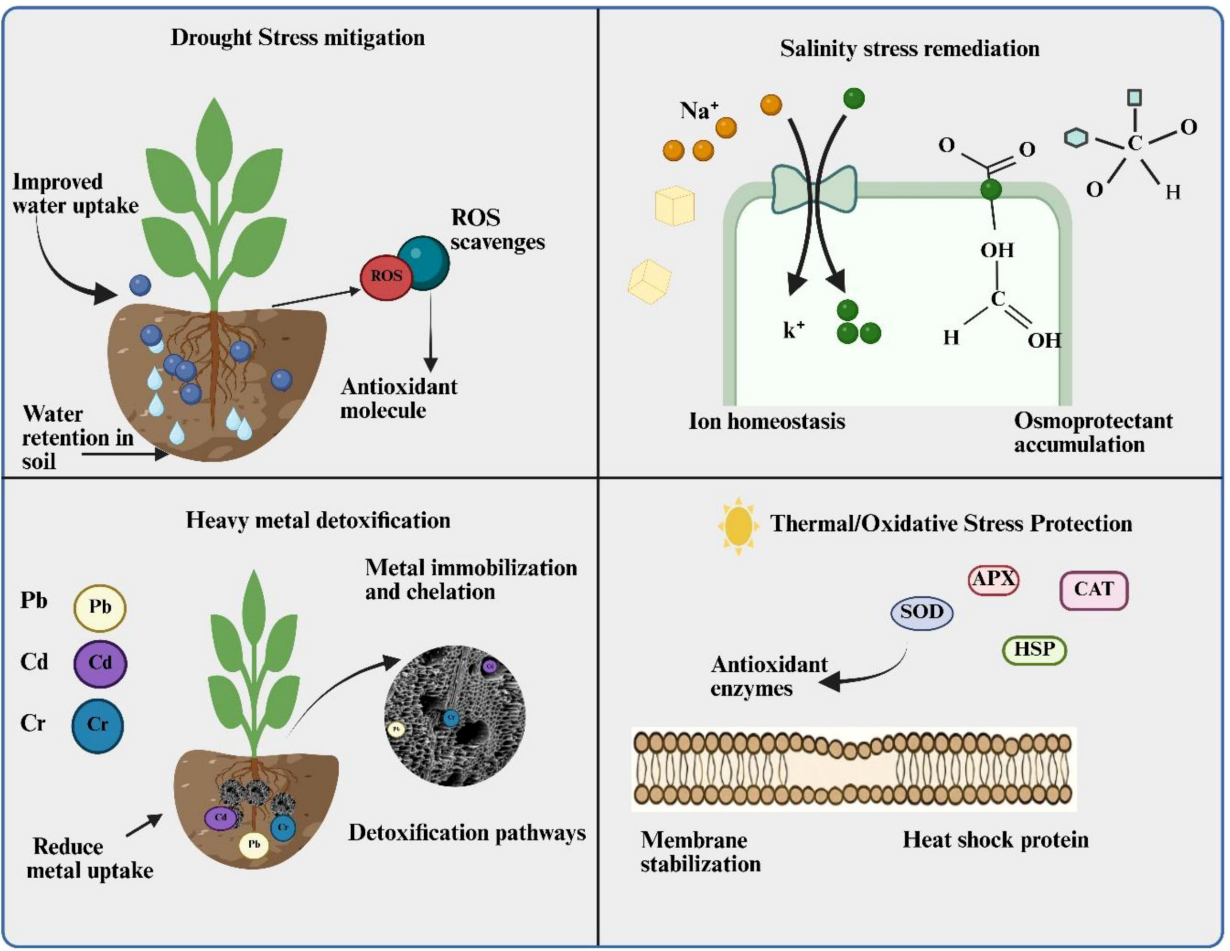
Mechanistic overview of biochar nanocomposite-mediated enhancement of plant resilience under abiotic stresses. EBNCs mitigate **(A)** drought stress by improving soil water retention, enhancing root water uptake, and scavenging ROS through antioxidant activity. **(B)** Under salinity stress, BNCs influence Na^+^/K^+^ balance and stimulate osmo-protectant accumulation to maintain ionic and osmotic homeostasis. **(C)** In heavy metal-contaminated soils, BNCs immobilize and chelate metals (Pb, Cd, Cr), reducing uptake and activating detoxification pathways. **(D)** During thermal and oxidative stress, BNCs enhance membrane stability and upregulate antioxidant enzymes (SOD, CAT, APX) and heat shock proteins (HSPs), protecting cellular structures. The image was created using BioRender (biorender.com).

Drought stress represents one of the most significant abiotic factor and large areas of the worlds agricultural regions have been exposed to this phenomenon, which is caused by reduced precipitation and limited water resources available for crops (Fahad et al., 2017; Murtaza et al., 2025). In recent years, due to more economic growth and global climate change, many agricultural production systems throughout the world have become susceptible to drought stress (Tang et al., 2019). This drought stress has significant impact on plant physiological, biochemical and molecular processes, disrupting plant-water relations at the cellular, tissue, and organ levels (Tang et al., 2019). When drought occurs, there is significant decline in chlorophyll content, relative water content and productivity of major crop plants (Bhargava and Sawant, 2013). EBNCs addition has been reported to improve soil water retention and is associated with enhanced antioxidant capacity under drought conditions (Farouk, 2025) as shown in **Figure 2**. The porous structure of BC and ability of certain NPs to absorb water develop an optimal moisture retaining environment that maintain soil moisture for longer period (Ali et al., 2021). Furthermore, the addition of metal oxide NPs (i.e. ZnO and TiO_2_) into BC has been shown to boost activity of various antioxidant enzymes including superoxide dismutase (SOD), catalase (CAT), and peroxidase (POD) (Sehrish et al., 2024). These enzymes protect cellular components by scavenging excessive damaging ROS while maintaining basal ROS levels required for stress signaling under drought stress (Sana et al., 2024).

It is important to distinguish between the dual roles of ROS in plant stress responses. At low to moderate concentrations, ROS function as essential signaling molecules that influence stress acclimation, stomatal closure, programmed cell death, and defense gene expression (Waszczak et al., 2018). This transient ROS burst, typically lasting minutes to hours, activates mitogen-activated protein kinase (MAPK) cascades and calcium-dependent pathways that enhance stress tolerance (Baxter et al., 2014; Mittler, 2017). However, under chronic or severe abiotic stress conditions, sustained ROS overproduction can exceed the cellular antioxidant capacity, leading to oxidative damage of lipids, proteins, and DNA, causing cellular dysfunction and programmed cell death (Qi et al., 2017; Noctor et al., 2018). The reported beneficial effects of EBNCs are associated with improved ROS homeostasis rather than complete ROS elimination, suggesting a balance between preservation of signaling ROS and scavenging of excessive damaging ROS (Zandalinas et al., 2018; Hasanuzzaman et al., 2020). However, direct temporal evidence demonstrating selective preservation of signaling ROS alongside suppression of chronic oxidative accumulation in EBNCs treated systems remains limited. Recent, studies showed that under drought stress the combined application of 0.75% nano-biochar (NBC) in soil and 120 mgL^-1^ brassinosteroids (BR) foliar spray significantly enhanced antioxidant defenses in wheat at tillering and anthesis in sandy loam soil, with SOD, POD, and CAT activities increasing by ~26%, ~19%, and ~15% compared with drought controls (Raza et al., 2023). BC-SiNPs significantly enhanced wheat performance by increasing antioxidant enzyme activities (SOD, CAT, POD, and APX by ~30–80%), improving photosynthetic efficiency, water status, and increasing grain yield compared with either BC or Si-NPs applied alone (Zulfiqar et al., 2024). However, BC-derived nanoparticles (BNPs) exhibited phytotoxic effects by inhibiting seed germination in rice and reducing shoot length and biomass in reed, depending on feedstock type and BC production temperature, highlighting potential dose- and crop-dependent environmental risks (Zhang et al., 2020). Under 40% field capacity drought stress in sandy loam soil, the combined application of rice husk BC (50 g kg^-1^ soil) and Fe_3_O_4_NPs (400 ppm) increased DPPH scavenging activity by ~8–9%, suggesting enhanced antioxidant capacity at the vegetative stage compared with drought stressed control plants (Sah et al., 2025). And slow release of micronutrients from these composites help in osmoregulation and response mechanisms in plants in stress (Hanif et al., 2024). In addition, Zn and Fe released from nanocomposite play a crucial role as cofactors in many enzymes and contribute to the plant’s cellular defense mechanisms (Ahmad et al., 2024). The gradual release of these micronutrients ensure the availability to plants and support under stress period, also minimize the risk of nutrient toxicity (Ghandali et al., 2024). Compared to this nano-BC with high concentrations can cause dose-dependent toxicity to soil microbes and crop nutrient uptake, highlighting potential ecotoxicological risks in agroecosystems (Rashid et al., 2023; Zhang, 2025). Experimental evidence indicates that EBNCs mediated mitigation under drought varies with stress severity (Rasheed et al., 2024). Under moderate drought conditions, synergistic effects on water retention, antioxidant activity, and growth are frequently reported. Under extreme or prolonged drought, however, physiological damage may exceed the buffering capacity of EBNCs mediated interventions. Collectively, these findings suggest that EBNCs performance operates within an operational stress window rather than providing uniform protection across all drought intensities.

### 4.2 Salinity stress: improving ion homeostasis and osmo-protection

Saline soils are characterized by an EC of more than 4 dS m^-1^ (≈40 mM NaCl) measured at 25 °C and have more than 15% exchangeable salts (Grigore and Toma, 2017). Above this threshold most crops show yield reduction. There are about 33% of irrigated lands and 20% of total cultivated lands in the world are salt affected (Negacz et al., 2022), and the affected area continues to expand annually due to climate change such as low rainfall, excessive evaporation, use of saline water for irrigation and poor soil management systems (Nachshon, 2018). Projections suggest that salinity may affect a larger fraction of arable land by 2050 if current trends persist. Soil salinity can disrupt plant physiological and biochemical processes through ionic imbalance and osmotic stress. Higher Na^+^ concentration leads to loss of cytosolic K^+^ and Ca²^+^, causing nutrient imbalance, oxidative stress and cell damage (Ahanger et al., 2015). Extreme higher saline condition will affect photosynthesis in plant through stomatal closure, degradation of chlorophyll molecule, enzyme inhibition and chloroplast disruption (Manzoor et al., 2025). Moreover, excessive amount of Na^+^/K^+^ and Na^+^/Ca²^+^ ratios inhibit ion uptake, disrupt cell membranes and decrease enzyme activity (Quintero et al., 2007). In addition to the above effects, salt stress triggers ROS accumulation that plays a dual role: initial ROS bursts activate salt overly sensitive (SOS) pathways and ion transporter regulation (Yang and Guo, 2018; Van Zelm et al., 2020), while prolonged exposure leads to chronic oxidative stress that disrupts macromolecules (proteins, nucleic acids, and lipids) and photoreceptors essential for plant growth (Munns and Tester, 2008). Over 800 million hectares of global agricultural land is affected by salinity that demanding some advanced mitigation strategies (Gao et al., 2024). Evidence indicates that under salinity stress EBNCs are associated with ion homeostasis, enhance osmo-protection, and improve the ability of plants to differentiate Na^+^ and K^+^ ions (Gill et al., 2024) shown in **Figure 2**. Comparative studies showed that Si-enriched BNCs reduce Na^+^ accumulation and enhance growth under salinity more effectively than individual components; for example, ZnONPs+BC significantly enhanced antioxidant enzyme activities in spinach at vegetative stage under 100 mM salinity, and elevated SOD (≈71–79%), POD (≈78–83%), CAT (≈74–87%), and APX (≈81–87%) activity with foliar application compared to control (Ahmad et al., 2024). BC with SiNPs and CaNPs lowered sodium uptake by ~32 % and increased safflower growth and seed yield by ~24.6 % and ~37 % under 6–12 dS m^-1^ salinity, while 200 mg L^-1^ SiNPs mitigated oxidative stress in tomato at seedling stage by boosting antioxidant enzymes and reducing H_2_O_2_ and MDA by ~41–42 % and ~45–50 %, respectively (Ghassemi-Golezani et al., 2024; Wang et al., 2025). BC matrices can limit harmful ion translocation; for example, BC-NPs reduced Na^+^ translocation from roots to shoots (TF~0.46) under salinity compared to controls (TF~0.72), indicating lower aboveground accumulation (Tao et al., 2023). Beside this, BC provides opportunity for cation exchange capacity that favor the soil buffering capacity against excessive salinity, while NPs contribute to specific ion regulating mechanisms (Soliman et al., 2023).

Biocarbon enriched with SiNPs and ZnNPs significantly enhances salt tolerance in different crops by improving K^+^/Na^+^ ratios and reducing Na accumulation in plant tissues (Ahmad et al., 2024). The nanocomposites play an important role in producing various compatible solutes such as glycine betaine, proline, and trehalose etc., which serve as osmo-protectants under salinity stress (Ayman et al., 2024; Gao et al., 2025). EBNC-associated increases in antioxidant enzyme activity are frequently accompanied by reduced H_2_O_2_ and MDA accumulation, suggesting improved redox balance under salinity stress (Abdelaal et al., 2020; Raza et al., 2023). Recent reviews indicate that NMs broadly associated with plant stress responses through modulation of redox signaling and defense activation; however, EBNC-specific temporal regulation of these pathways remains to be systematically validated (Chadha et al., 2024). Although BC and BC–NPs composites mitigate salinity stress, responses remain strongly dependent on dose, soil type, crop genotype and salinity stress because over dose or inappropriate application can negatively impact soil microbes, plant growth, and water relations (Rasheed et al., 2024; Zhang, 2025). Biogenic ZnONPs significantly reduced soil microbial biomass carbon by 27- 43% at higher dose, and rice-straw derived BC did not alleviate this transient toxicity in calcareous soil (Shemawar et al., 2021). The incorporation of BC and graphene oxide significantly increases surface area and introduces large number of functional groups, improving the overall adsorption capacity and ion exchange properties of these composite material (Du et al., 2020). These enhanced physicochemical characteristics are associated with improved ion adsorption and buffering capacity, which may contribute to improved plant performance under defined salinity conditions. Similarly, EBNCs efficacy under salinity appears to follow a threshold-dependent pattern. At lower salinity levels, intrinsic plant tolerance mechanisms may limit observable material-associated gains, whereas under moderate salinity, improvements in Na^+^ partitioning, K^+^/Na^+^ ratios, and redox balance are continually reported. Under severe salinity or excessive nanoparticle loading, however, benefits may plateau or decline, reflecting physiological saturation and potential secondary stress effects. In such cases, where ionic toxicity and osmotic stress intensify beyond physiological compensation thresholds, buffering effects may be insufficient to prevent functional impairment, indicating the presence of an operational stress window (Munns and Tester, 2008; Rasheed et al., 2024).

### 4.3 Heavy metals stress: immobilization, chelation, and detoxification

Pollution of HMs implies excessive accumulation of toxic metals such as cadmium (Cd) lead (Pb), chromium (Cr), mercury (Hg), and arsenic (As) beyond permissible limits that causes serious damage to ecosystems and biodiversity, and threatening human health (Nagajyoti et al., 2010). These toxic metals mainly comes from agricultural, industrial and urban activities (Ali et al., 2013). Plants are considered the first affected community, this occur when plants absorb metals through their roots and then translocated to shoots and productive parts, thereby causing reduction in nutrient uptake, disrupt certain metabolism, and decrease the plant growth (Song et al., 2017). HMs toxicity is intricately linked with ROS-mediated oxidative stress, where metals like Cd, Pb, and As induce both signaling ROS that activate metal detoxification pathways and damaging ROS that cause lipid peroxidation and membrane damage (Cuypers et al., 2016; Singh et al., 2016). Hence, HMs contamination poses severe threat to agricultural productivity and food safety. EBNCs offers versatile approaches including chelation, immobilization and detoxification mechanisms to mitigate HM stress (Aborisade et al., 2023) as represented in **Figure 2**. The high surface area and various functional groups associated with BC provide adsorption sites for HMs, while NPs provide additional binding mechanisms (Manzoor et al., 2022b; Khalid et al., 2023). Application of Fe-BC nanocomposites has been shown excellent performance for the immobilization of Cd and Pb due to formation of stable complexes of metal-oxide and precipitation reactions through series of interactions (Latifian et al., 2024). Beyond physical immobilization, EBNCs mitigate HM-induced oxidative stress by maintaining ROS balance enabling activation of metal stress response genes while preventing oxidative injury to cellular components (Rajput et al., 2021). The superiority of EBNCs over individual components is particularly evident in HM remediation. BC-supported nZVI composites stabilized Cd, reducing leaching to 8.23 mg/L (BH4-nZVI/BC) and 4.65 mg/L (TP-nZVI/BC; 30–61% improvement), with TP-nZVI/BC showing long-term stability and enhanced microbial diversity. Likewise, Fe-loaded BC lowered Cd in rice grains and roots by 68% and 48%, reduced soil exchangeable/acid-soluble Cd by up to 64%, increased pH by 0.53 units, and enhanced Fe oxide content, demonstrating synergistic Cd immobilization in the rhizosphere (Sui et al., 2024; Jia et al., 2025). However, saturation effects limit HM immobilization at high contaminant loads. The magnetic characteristics of FeNPs facilitate recovery and recycling of contaminated materials (Aborisade et al., 2023) and also serve as nano-fertilizer for sustainable plant production (Manzoor et al., 2022a, 2023) under stress environment. Recent studies have shown that Fe-BC composites can decrease about 85% cadmium availability in soil (Ali et al., 2024) and BC-nano Si reduced the As in roots by 61% and in straw by 37% of *Capsicum annuum* L. then control group without product (Manzoor et al., 2024). The incorporation of CaNPs and MgNPs into the BC can bind HMs through producing effective chelation systems and reduced their uptake in plants (Ghandali et al., 2024). Furthermore, these nanocomposites also provide distinct essential nutrients that enhance the growth and stress tolerance mechanisms in plants. Along with this, pH buffering capacity of these composites also increases HM immobilization through complexation and precipitation (Kochar, 2024). For HMs stress, EBNCs demonstrate the greatest advantage under moderate contamination levels where extracellular immobilization and intracellular detoxification mechanisms can meaningfully reduce uptake. At very low contamination levels, effects may be negligible, whereas under extremely high metal concentrations, phytotoxicity may surpass the sequestration capacity of the composite, indicating a bounded remediation window.

### 4.4 Thermal and oxidative stress: antioxidant enhancement

Both extreme temperature and oxidative stress often occur simultaneously that require comprehensive defense strategies (Rahim et al., 2025). The distinction between beneficial signaling ROS and harmful chronic ROS accumulation is particularly critical under thermal stress, where initial ROS bursts trigger heat shock protein expression and acquired thermotolerance, while prolonged exposure leads to oxidative injury (Suzuki et al., 2012; Mittler et al., 2022). EBNCs will enhance plant tolerance against thermal stress through improved antioxidant systems, stabilization of cellular structures and improved heat dissipation (Abdelhameed et al., 2025) as observed in **Figure 2**. Along with this the thermal properties of BC also provide insulation effects, which can moderate soil temperature variations (Rafique et al., 2025). BC is able to blend metal NPs with BC as catalyst for antioxidant enzyme reactions. As metal NPs function as catalyst, they can enhance the efficiency and speed of the ROS scavenging from plant cells (Sehrish et al., 2024). ZnNPs appears to be effective at enhancing antioxidant properties and supporting the cellular defense mechanisms under conditions of thermal stress (Jafir et al., 2024). The controlled release of these metals from embedded BC ensures continuous supply of antioxidant protection throughout the stress period. Carbon-based nanomaterials such as graphene nanoplatelets and multi-walled carbon nanotubes combined with BC have also been shown to scavenge ROS effectively through enhanced antioxidant systems. And these materials enhanced plant antioxidant activity up to 184% when exposed to stress conditions (Jafir et al., 2024). These mechanisms may contribute to reduce oxidative damage under environmental stress conditions.

### 4.5 Convergence of stress pathways: unique co-regulation by EBNCs

Unlike BC or NPs applied individually, EBNCs have been reported to influence multiple interconnected stress pathways, potentially generating defense responses beyond those achieved with single components. They are associated with improved ROS homeostasis, permitting transient signaling bursts involved in pathway activation (e.g., MAPK, Ca²^+^-SOS, DREB2A/WRKY), while enhanced antioxidant enzyme activities (SOD, CAT, APX), often increasing by 40–70%, may limit chronic oxidative damage (Waszczak et al., 2018; Aborisade et al., 2023; Ahmad et al., 2024). EBNC-mediated improvements in antioxidant capacity may contribute to stabilization of ROS-dependent stress signaling pathways; however, whether this represents a diverse developing systems-level, coordination mechanism remains to be rigorously validated across crop–soil systems. Under salinity, EBNCs have been shown to reduce plant sodium accumulation and enhance ionic homeostasis by enhancing soil cation exchange, limiting Na^+^ uptake, and supporting vacuolar Na^+^ sequestration, thereby contributing improved nutrient balance and osmotic adjustment more effectively than single components; for example, BC-based NCs lowered leaf Na^+^ by ~22–26 % in dill (6–12 dS m^-1^ salinity), and BC significantly upregulated NHX1 expression (≥2-fold) in Chinese cabbage (Ghassemi-Golezani and Rahimzadeh, 2023; Rathinapriya et al., 2025). For HMs, For heavy metals, EBNCs integrate extracellular sequestration, apoplastic barrier reinforcement, and intracellular detoxification processes, with some studies reporting reductions in grain Cd accumulation of ~40% compared with untreated controls (Manzoor et al., 2021). EBNCs application has also been associated with priming of systemic immunity, enhancing ROS-mediated defense signaling while maintaining relatively low growth penalties in certain crop systems (Kong et al., 2022). Collectively, these findings suggest that EBNCs may facilitate coordinated, multi-tiered stress regulation under defined experimental conditions; however, the reproducibility and generalizability of such systems-level integration across diverse agroecosystems requires further mechanistic validation. A comprehensive summary of the mechanisms mediated by EBNCs and their applications across different abiotic stresses is presented in **Table 1**.

## 5 Mechanisms of EBNCs in mitigating biotic stress

### 5.1 Pathogen suppression: antimicrobial properties of metal-loaded biochar

**Figure 3:**
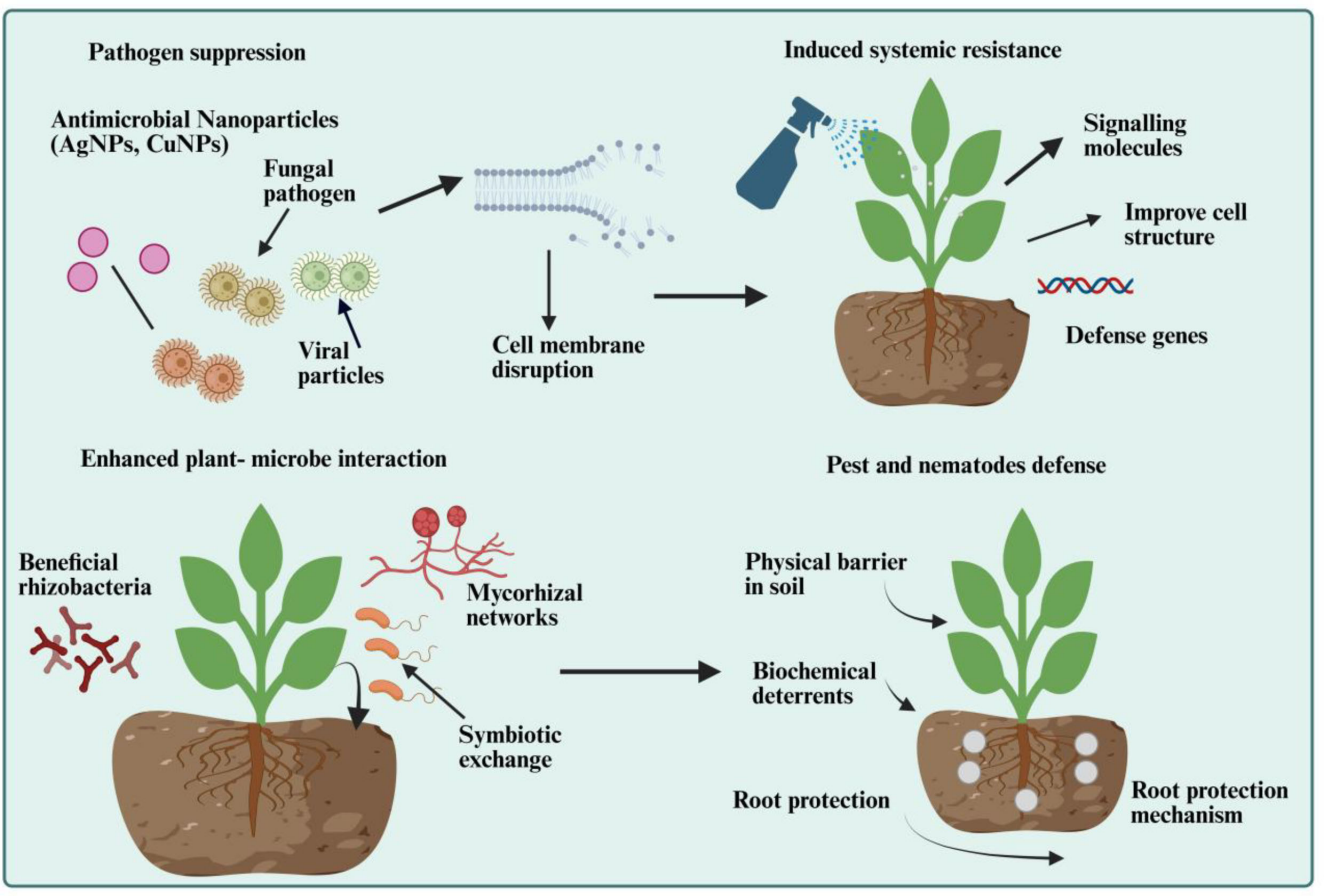
Multifunctional roles of EBNCs in enhancing plant defense against biotic stresses. Biochar-based nanocomposites containing antimicrobial nanoparticles (e.g., AgNPs, CuNPs) suppress pathogens by disrupting fungal and viral cell membranes, thereby reducing infection and disease incidence. BNCs also trigger induced systemic resistance (ISR), enhancing cell structure integrity, and upregulating defense-related genes. Furthermore, they promote beneficial plant–microbe interactions, stimulating rhizobacterial colonization and mycorrhizal symbiosis, which enhance nutrient exchange and plant immunity. In addition, BNCs provide pest and nematode defense through the formation of a physical barrier in soil, release of biochemical deterrents, and reinforcement of root protection mechanisms. The image was created using BioRender (biorender.com).

Plant diseases cause substantial agricultural losses annually, necessitating effective and sustainable control strategies. EBNCs can combat pathogenic microorganisms directly through controlled release of metal ions, ROS generation, and disruption of pathogen cell’s metabolic systems (Zhang et al., 2024) as shown in **Figure 3**. Ag-loaded BC exhibits excellent antimicrobial efficacy against bacterial, fungal, and viral pathogens without plant productivity (Nworie et al., 2024). However, it is critical to distinguish between pathogen suppression effects and pesticidal claims; while EBNCs demonstrate antimicrobial properties in controlled conditions, their field efficacy should not be overstated as equivalent to registered pesticides without rigorous dose-response studies, spectrum analysis, and regulatory evaluation (Adisa et al., 2019; Kah et al., 2019). The antimicrobial mechanisms of metal NPs in EBNCs include protein denaturation, DNA damage, cell membrane disruption and disruption of cellular respiration (Hassan et al., 2025). The sustained release from BC matrices ensures prolonged antimicrobial activity while reducing potential for resistance (Viswanathan et al., 2024). Recent studies have found that Ag-BC NCs suppress the growth of pathogens by 90% with least adverse effect on beneficial soil microorganisms (Irshad et al., 2024). CuNPs and ZnNPs in BC have demonstrated antimicrobial activity across a wide spectrum with additional nutritional benefits for plants (Mazhar et al., 2024). These nanocomposites have been shown to control a distinct variety of pathogens effectively such as *Fusarium*, *Pythium* and *Xanthomonas* species (Mahmoud et al., 2024; Mazhar et al., 2024). The dual function of controlling plant pathogens and supplying nutrients may enhance the value of these materials for integrated plant health management.

### 5.2 Induced systemic resistance: priming plant immune responses

EBNCs can activate ISR in plants, thus providing them with greater natural defense mechanisms against different biotic stresses (**Figure 3**). This priming effect involves activation of defense gene expression, enhance accumulation of antimicrobial substances, and increased sensitivity to pathogen-associated molecular models (Zhang et al., 2024; Kalia and Sreelakshmi, 2025). BC-NPs have been reported to induce ISR responses and may contribute to broad-spectrum resistance against certain pathogens (Arshad, 2024). These EBNCs induced various ISR mechanisms through activating signaling pathways of salicylic acid and jasmonic acid, increased phytoalexins production, and enhanced activity of defense-related enzymes such as chitinase, phenylalanine ammonia-lyase and β-1,3-glucanase (Mushtaq et al., 2024). These responses create a primed state in which plants can respond rapidly and effectively to pathogen threats. The size and surface properties of NPs play a key role in influencing their ability to induce ISR responses. The most effective size range of NPs for inducing ISR is 20–50 nm, while surface functionalization with specific molecules can improve recognition by plant immune systems (Kalia and Sreelakshmi, 2025). ISR activation improves pathogen resistance but is intrinsically linked to a growth–defense trade-off in immune-primed plants. Constitutive or chronic activation of ISR pathways diverts photosynthetic resources and carbon allocation from growth and reproduction to defense metabolism, potentially reducing crop yields under non-stressed conditions (Huot et al., 2014). Sustained immune activation often leads to yield penalties as metabolic resources are diverted from growth toward defense, particularly in environments with minimal pathogen challenge (Ning et al., 2017). Therefore, optimal EBNC application strategies should aim for transient priming rather than sustained immune activation, balancing enhanced pathogen readiness with minimal fitness costs (Conrath et al., 2015). By controlling the way through which NPs are presented to the plants roots, the BC matrix optimized the ISR instigation (Deng et al., 2023). The efficacy of EBNC induced ISR is highly context-dependent, influenced by pathogen pressure, environmental conditions, and crop developmental stage. EBNCs are most beneficial when applied prophylactically in pathogen-prone environments or during vulnerable growth stages (e.g., seedling establishment), where the benefits of enhanced immunity outweigh metabolic costs (Walters et al., 2013; Martinez-Medina et al., 2016). However, excessive or poorly timed activation of plant defense responses may impose physiological costs, including growth retardation, due to heightened immune signaling and resource reallocation (Liang et al., 2022). Dose-response studies have documented that higher concentrations of NPs or nanocomposites in soil can trigger defense overstimulation and phytotoxicity, with effects dependent on particle type, soil properties, and plant species (Mirbakhsh, 2023). Consequently, precision application and site-specific optimization are necessary to leverage ISR benefits while avoiding detrimental overactivation of plant immunity.

### 5.3 Enhanced plant–microbe interactions: supporting beneficial rhizobiota

EBNCs can enhance beneficial plant-microbe interactions while inhibiting the growth of pathogenic organisms (**Figure 3**). In this composite BC provides habitat and supply distinct types of nutrients to beneficial microorganisms, while NPs contribute to selective antimicrobial activity (Chandi et al., 2024). This selective effect of nano-BC supports the development of healthy rhizosphere populations that promote plant health and stresses tolerance (Elhakem et al., 2025) against various stresses. In addition, using BC with specific types of NPs (i.e. ZnO and Fe) to the rhizosphere can promote nitrogen fixation by rhizobia, the establishment of mycorrhizal fungi, and the activity of growth promoting bacteria. These interactions contribute to improved nutrient acquisition, stress tolerance, and overall plant health (Haider et al). The sustained release of essential micronutrients from EBNCs supports microbial activity and communication between the plant and associated microorganisms. Recent research has shown that use of EBNCs can increases beneficial microbial populations by 2 to 3 times and reduced the pathogenic microorganisms, creating a balanced rhizosphere environment (Tikoria et al., 2023). The spatial arrangement of NPs within the BC matrix influences the composition and function of the microbial community in the environment. This modulation of microbial communities represents a sustainable method of managing plant health (Minello et al., 2025). Despite the benefits of enhanced plant-microbe interactions, careful consideration must be given to potential ecosystem risks. Nano formulations may impact non-target organisms, and uncontrolled release of active compounds could influence soil microbial dynamics and functions (Grillo et al., 2016). Several studies showed that ENPs can adversely affect soil microbial communities; Ag nanomaterials reduce arbuscular mycorrhizal colonization, while metal oxide NPs such as TiO_2_NPs can disrupt nitrifying microbial communities and impair soil microbial functions (Judy et al., 2015; Simonin et al., 2016). Long-term assessment of soil biological responses is important to ensure that stress-ameliorating strategies, including EBNCs, do not unintentionally compromise rhizosphere functioning and plant–microbe interactions (Meena et al., 2022).

### 5.4 Pest and nematode defense: physical and biochemical barriers

EBNCs provide defense against insects and plant-parasitic nematodes through physical and biochemical mechanisms (**Figure 3**). The physical barrier comes from some NPs being abrasive and the modification of plant surface properties (Zhang et al., 2024). Biochemical effects include enhanced production of defensive compounds and disruption of pest cellular processes (Irshad et al., 2024). Silica NPs, when integrated into BC, provide an effective pest management system. Their insecticidal action works through direct mechanical abrasion of the cuticle, leading to desiccation, and indirectly by blocking the insect’s digestive tract upon ingestion (Thabet et al., 2021). Iron oxide nanoparticles (FeONPs) showed effectiveness against root-knot nematodes through multiple mechanisms including physical disruption and production of ROS (Mahmoud et al., 2024). The controlled release of bioactive compounds from EBNCs can repel or kill insect pests without affecting beneficial insects (Jing et al., 2025). These BC-NCs may provide sustained pest control while reducing environmental impacts compared with conventional pesticides. A comprehensive summary of EBNCs-mediated mechanisms and outcomes in managing biotic stresses is presented in **Table 2**.

**Table 2:** Biochar-based nanocomposites for managing biotic stresses: mechanisms and outcomes.

Nanocomposite type	Plant / target system	Proposed mechanism	Key findings / outcomes	Reference
Green FeONPs + magnetic nano-BC	Tomato / root-knot nematode	BC–FeO nanocomposite increases NP stability, Fe availability, and plant defense signaling, leading to plant-mediated nematode resistance.	Improved tomato growth, and phytochemical accumulation, with reduced root-knot nematode infestation/ population	(Mahmoud et al., 2024)
Biogenic waste-derived colloidal/nanobiochar	Colletotrichum gloeosporioides (fungal pathogen)	Growth inhibition via surface-mediated interactions of nano-BC with fungal hyphae, leading to reduced mycelial density and pathogenicity	Reduced in mycelial densities and inhibited fungal growth and effectively control anthracnose disease	(Nishshankage et al., 2024)
BC + zinc oxide NPs	Vigna radiata (mung bean) / charcoal rot (Macrophomina phaseolina)	Synergistic disease suppression via ZnO-NP mediated antifungal activity and BC-induced improvement in soil health and plant antioxidant defense, reducing oxidative stress and pathogen impact.	Significantly reduced charcoal-rot disease severity, enhanced antioxidant enzyme activity, and improved growth, yield, and physiological performance of Vigna radiata.	(Mazhar et al., 2024)
Engineered BC + fly ash NPs	Pepper / bacterial leaf spot	Surface-mediated antibacterial activity and induced plant defense responses limiting pathogen proliferation.	BC and NPs significantly reduced bacterial leaf spot severity and enhanced pepper growth.	(Aftab et al., 2022)
BC-ZnO nanocomposite (MB-ZnO)	Kiwi (Actinidia deliciosa) / brown leaf spot	BC-ZnO nanocomposite exhibits surface-mediated antifungal activity that interacts with Rhizopus oryzae, disrupting fungal growth and inhibiting pathogen proliferation.	MB-ZnO significantly inhibited R. oryzae mycelial growth (up to ~79% at 19 mg/mL), suggesting effective eco-friendly control of brown leaf spot in kiwi	(Kamal et al., 2023)
Cordyceps fumosorosea- BC-NPs	Survival of Bemisia tabaci (Gennadius)	BC-NPs enhance fungal infection, leading to egg desiccation and immature mortality through pathogenic interaction with whitefly life stages	Increased mortality and reduced hatchability and survival of B. tabaci eggs and immatures with dose-dependent effectiveness of C. fumosorosea-BC NPs.	(Wang et al., 2021)
BC NPs + methoxyindole	Nicotiana benthamiana	BC-NPs provide resistance to P. nicotianae infection by increasing ROS, activating ethylene pathway and induced systemic acquired resistance expression	Enhanced immunity through stimulating plant defense responses, reduced pathogen damage and enhanced plant growth	(Kong et al., 2022)
Cu-based NPs + rice husk ash	Fungal pathogens (various)	Copper-based NPs disrupt fungal growth and enhance plant defense responses, with RHA acting as carrier and stabilizer	Significant reduction in fungal growth and disease incidence, stimulate crop growth and phytohormones production	(Tran et al., 2025)
Bimetallic NPs + BC from Adansonia digitata shell	Tomato / fungal pathogens	Bimetallic Ag-FeO NPs disrupt fungal cell walls and inhibit growth via direct NP–pathogen interaction, with BC serving as a green support matrix.	Ag and Ag-FeO NPs significantly reduced growth of tomato pathogenic fungi; BC alone showed no direct antifungal effect	(Aldahasi et al., 2024)
nanocomposites of BC, attapulgite (ATP), carboxymethyl chitosan (CMCS), and gelatin (Gel)	Cabbage / Aphid infestation	BNCs enables pH/temperature-responsive protection and controlled release of thiamethoxam, reducing non-target dispersion while enhancing localized insecticidal efficacy.	Stimulus-responsive control releases improved environmental stability and aphid control, providing prolonged protection with reduced off-target loss.	(Jing et al., 2025)

## 6 Challenges and considerations in EBNC implementation

Despite their expected benefits, concerns regarding the potential risks associated with EBNCs have increased, highlighting the need for careful monitoring and continuous evaluation. A key risk of EBNCs is the potential leaching of engineered NPs from the BC matrix into the environment, where their accumulation may adversely affect non-target organisms (Atanda et al., 2025). Therefore, careful consideration of the long-term fate of engineered NPs in soil systems is essential to ensure their safe use within the ecosystems. Different types and concentrations of engineered NPs exhibit varying toxicity levels; for example, although AgNPs are highly effective against bacteria, certain doses can harm beneficial soil microbes (Nworie et al., 2024). Therefore, to ensure the safe environmental application of EBNCs, researchers must design materials that balance desired antimicrobial efficacy with ecological safety. Researchers may develop controlled-release mechanisms and systematic approaches toward dosing, to provide assurance that EBNC’s will not adversely impact the environment. Current studies indicate that engineered NPs with surface functionalization and stabilization techniques can reduce their toxicity level (Viswanathan et al., 2024). Further, use of green synthesis methods approaches and biodegradable coating materials can significantly reduce environmental risks while maintaining functional properties (Kalidasan et al., 2025). Additionally, one of the most critical dimensions of ensuring EBNCs arrive safely to market is the need for standard methods for determining the toxicity of engineered products. EBNCs can involve trade-offs regarding increased crop productivity and decreasing crop production due to potential longer-term environmental effects. Research will be required on the impact of EBNCs on the soil microbial community, the cycling of nutrients, and ecosystem performance, as they improve the plant’s ability to increase yields and adapt to environmental stress (Mushtaq et al., 2024). Additional risk-benefit analyses should also include aspects of bioaccumulation through the food chain and the potential environmental impacts on soil biodiversity caused by the use of EBNCs.

The effectiveness of nanomaterial-based soil amendments is constrained by saturation, competition, and environmental conditions. Fe-modified BC’s exhibit finite Cd sorption capacities, with immobilization efficiency declining as adsorption sites become occupied (Park et al., 2013), while competitive interactions in multi-metal systems can reduce Pb immobilization by up to ~40% (Han et al., 2017). Moreover, NP performance is temporally and chemically sensitive: AgNPs lose antimicrobial activity through sulfidation and passivation in soils (He et al., 2019), and soil pH strongly influence metal NP dissolution and mobility under alkaline and acidic conditions (Chen et al., 2019; Shemawar et al., 2021).

Soil health indicators respond differently to various EBNC formulations, with some formulations enhancing beneficial microbial activity and some temporarily disrupting beneficial microbial activity. The timing and application method can also have significant influence on environmental outcomes (Tikoria et al., 2023). Therefore, regarding sustainable implementation of EBNCs for longer period, it is essential that nutrient cycling, soil biological activity, and ecosystem services are monitored (Liang et al., 2025). The interaction between EBNCs and existing soil amendments, fertilizers, and pesticides may result in unexpected environmental consequences. So, comprehensive studies are required to quantify the interactions to develop compatible integrated management systems (Yang et al., 2025). Upon repeated application of EBNC, special consideration is required regarding cumulative impact of applying EBNCs to prevent environmental degradation. To be commercially successful, manufacturers of EBNCs need to produce EBNCs at a price point that allows for mass adoption. Currently, the production costs often exceed those of conventional amendments, making them inaccessible to small-scale farmers (Zhou et al., 2024). Therefore, the development of scalable synthesis methods and utilization of low-cost feedstocks are critical for successful commercialization. During developing scalable synthesis methods, it is important to consider challenges related to quality control, standardization of the synthesis process, and the ability to maintain performance characteristics throughout large-scale production (Velasquez‐Pinas et al). Due to complexity associated with EBNC synthesis, some manufacturers may require specialized equipment as well as skilled labor to produce these nanocomposites; therefore, making production methods to implement and produce, while maintaining quality and performance characteristics, represents a major research priority. Barriers regarding farmer adoptions includes lack of awareness, required initial investment, technical complexity and regulatory uncertainty (Azmir and Mohamed, 2025). Educational, demonstration, and technical support programs will greatly aid in transferring technology to local farmers. Furthermore, the development of user-friendly application methods and clear performance guidelines can facilitate adoption (Patra and Coumar, 2023).

The existing regulatory structure surrounding the use of nano-enabled agricultural amendments is poorly defined, causing a lack of clarity for researchers, producers or users of these NPs in agriculture. As current regulatory structure may not adequately address the distinctive properties of EBNCs and their associated potential risks, additional requirements must be established regarding specific guidelines and safety standards (Kansotia et al., 2024; Atanda et al., 2025). The international harmonization of regulatory methods is essential for global market development. Safety assessment protocols for EBNCs require adaptation of existing methods to account for the complex interactions between nanomaterials, BC, and agricultural systems (Chandi et al., 2024). Standardized procedures for environmental fate, toxicity, and efficacy need evaluation development and validation (Elhakem et al., 2025). For establishing acceptable exposure limits and safety margins there is need of wide range research as well as stakeholder consultation. Labeling and tracking nano-enabled materials present additional challenges for manufacturers and regulators. Requirements to assist users with clear identification of NPs content, potential hazards and guidance regarding safe application remain critical for productive use of EBNCs (Haider et al). Implementation of cultural practices to ensure the safe and effective use of EBNCs can be aided through the development of certification programs and quality assurance systems.

## 7 Conclusion and future prospects

This review synthesizes rational design principles for EBNCs, emphasizing how matrix-NP interactions such as BC pore architecture and surface chemistry-govern NP retention, mobility and release behavior, thereby enabling more predictable formulation of stress-specific composites (Qin et al., 2025). Feedstock–NP complementarity including lignin-rich BC for HM immobilization, ash-rich for nutrient release, Si-rich for stress tolerance enhancement, provides a framework for maximizing synergistic functionality (Aborisade et al., 2023; Ahmad et al., 2024). Importantly, it is necessary to distinguish between passive controlled release resulting from adsorption–desorption equilibria and pore confinement, and truly stimuli-responsive systems incorporating stress-labile chemical linkages (e.g., pH-, ROS-, or enzyme-sensitive bonds) (Jing et al., 2025). While most currently reported EBNCs rely on passive release mechanisms, future development of engineered stimuli-responsive architectures may enable more dynamic stress-adaptive activation. Multi-component EBNC formulations integrating diverse NP types onto a single BC matrix offer opportunities to address complex stress environments, provided that total NP loading is carefully optimized to avoid pore blockage and impaired transport functions (Rajput et al., 2021). Surface functionalization strategies may further enhance pathway selectivity (Kah et al., 2019), however, crop- and soil-specific validation remains limited. Together, these principles provide a mechanistic, design-oriented framework for EBNC development and validation across diverse agroecosystems.

Accumulating evidence suggests that EBNCs can outperform conventional BC under specific stress intensities and soil conditions. Reported mechanisms include improved water retention, enhanced antioxidant capacity, modulation of reactive oxygen species (ROS)-related pathways, antimicrobial effects, and induction of plant defense responses. However, these benefits are strongly context-dependent and vary according to crop species, soil chemistry, nanoparticle type, and application rate. Importantly, EBNC performance is not universally superior. Under mild stress conditions, conventional BC may provide comparable improvements in soil structure and water retention without the added complexity of nanoparticle incorporation. Conversely, under extreme stress intensities or excessive NP loading, sorption site saturation, competitive ion displacement, pore obstruction, or microbial community disruption may reduce efficacy or introduce unintended trade-offs. In certain soil systems, particularly calcareous or highly buffered soils, NCs advantages may be attenuated. These counterexamples indicate that EBNC efficacy is conditional rather than intrinsic and must be evaluated within defined environmental and stress-intensity boundaries. Moreover, long-term field-scale validation remains limited, and much of the current evidence derives from short-duration greenhouse or controlled-environment studies. Standardized stress-intensity thresholds and dose–response relationships are rarely reported, limiting cross-study comparability and predictive scalability. Comprehensive safety assessment frameworks addressing nanotoxicity, persistence, transformation (e.g., sulfidation or dissolution), and impacts on soil microbial networks remain essential prerequisites for responsible implementation.

Future research should prioritize (i) mechanistic validation of proposed emergent properties, (ii) identification of stress-intensity thresholds that define when EBNC–BC synergy emerges and when saturation or toxicity may dominate, (iii) long-term ecotoxicological and agronomic field evaluations, and (iv) scalable, cost-effective, and environmentally responsible synthesis strategies. Integration of multi-omics approaches with data-driven modeling and machine learning may assist in optimizing formulation parameters tailored to crop–soil–stress combinations, but such approaches require robust empirical datasets to ensure reliability and transparency. Circular production pathways utilizing agricultural residues and industrial by-products offer promising routes for sustainable EBNC fabrication, potentially improving resource efficiency while contributing to carbon management. However, economic feasibility, lifecycle assessment, and nanoparticle recovery or stabilization strategies must be rigorously evaluated before widescale deployment can be justified. Overall, EBNCs represent a developing class of multifunctional soil amendments with demonstrated potential to enhance plant resilience under defined environmental and stress-intensity conditions. Their successful translation from experimental systems to practical agriculture will depend on mechanistic clarity, stress-intensity–dependent performance validation, long-term safety assessment, and context-specific design optimization. A cautious, evidence-driven development pathway will be essential to determine where and when EBNCs provide measurable advantages over existing soil management strategies.
